# High circ-SEC31A expression predicts unfavorable prognoses in non-small cell lung cancer by regulating the miR-520a-5p/GOT-2 axis

**DOI:** 10.18632/aging.103264

**Published:** 2020-06-04

**Authors:** Mingming Jin, Chunzi Shi, Qian Hua, Tian Li, Chen Yang, Yue Wu, Licong Zhao, Hao Yang, Jiaqi Zhang, Cheng Hu, Gang Huang

**Affiliations:** 1 Shanghai University of Traditional Chinese Medicine, Shanghai University of Medicine and Health Sciences, Shanghai 201203, P.R. China; 2 Shanghai Key Laboratory of Molecular Imaging, Shanghai University of Medicine and Health Sciences, Shanghai 201318, P.R. China; 3 Department of Nuclear Medicine, Renji Hospital, School of Medicine, Shanghai Jiaotong University, Shanghai 200127, China; 4 Department of Urology, Huashan Hospital, Fudan University, Shanghai 200040, China; 5 China Medical University, Shenyang 110011, Liaoning, China; 6 Experiment Center for Science and Technology, Shanghai University of Traditional Chinese Medicine, Shanghai 201203, China

**Keywords:** non-small cell lung cancer, hsa_circ_0001421 (circ-SEC31A), miR-520a-5p, GOT-2, proliferation

## Abstract

Dysregulation of circular RNAs (circRNAs) has recently been shown to play important regulatory roles in cancer development and progression, including non-small cell lung cancer (NSCLC). However, the roles of most circRNAs in NSCLC are still unknown. In this study, we found that hsa_circ_0001421 (circ-SEC31A) was upregulated in NSCLC tissues and cell lines. Increased circ-SEC31A expression in NSCLC was significantly correlated with malignant characteristics and served as an independent risk factor for the post-surgical overall survival of NSCLC patients. Reduced circ-SEC31A expression in NSCLC decreased tumor cell proliferation, migration, invasion, and malate-aspartate metabolism. Mechanistically, we demonstrated that silencing circ-SEC31A downregulated GOT-2 expression by relieving the sponging effect of miR-520a-5p, which resulted in significantly reduced malate-aspartate metabolism in NSCLC cells. Taken together, these results revealed the important role of circ-SEC31A in the proliferation, migration, invasion, and metabolic regulation of NSCLC cells, providing a new perspective on circRNAs in NSCLC progression.

## INTRODUCTION

Lung cancer is a high-risk tumor that is the most common malignancy in the world and seriously endangers human health. It is also the most important factor in cancer-related deaths [1, 2]. Lung cancer accounted for 18.4% of all cancer deaths (equal to 1.8 million deaths) in 2018, with an age-standardized mortality rate of 18.6 (27.1 in men, 11.2 in women) per 100,000 persons, and an estimated 1.6 million deaths each year [3]. Accounting for approximately 85% of all lung cancers, non-small cell lung cancer (NSCLC) is the most common type of lung tumor, including squamous cell carcinoma, adenocarcinoma, and large cell carcinoma [4]. Lung cancer often has no obvious symptoms in the early stage, as its symptoms and signs appear later, so most patients are in middle-late stages at the time of treatment, and most have local spread or distant metastasis. The overall 5-year survival rate is approximately 15%, while for patients with distant metastasis, the 5-year survival rate is <4% [5].

Circular RNAs (circRNAs) are a special type of non-coding RNA that usually do not encode proteins and exist in any genomic region. The recent development of high-throughput sequencing technology and bioinformatics [6] has allowed a large number of endogenous circRNAs to been found in mammals [7]. Recent studies on circRNAs have led to the recognition that transcripts of many human genes can be reverse-cleared, nonlinearly [7] or by gene rearrangement [8], to form circRNAs. Moreover, circRNAs are abundant in all transcripts, structurally stable, sequence-conserved, and have the characteristics of tissue- and time-specific expression [9–14]; thus, they have good potential as biomarkers. At the same time, it was found that circRNAs play important roles in proliferation, apoptosis, invasion, and metastasis in various tumor cell types, including bladder cancer [15], liver cancer [16], colorectal cancer [17], gastric cancer [18, 19], and pancreatic cancer [20]. CircRNAs play important roles in regulating mRNAs at the transcriptional and/or post-transcriptional level: circular intronic RNAs or intronic sequence exon-intron circRNAs are primarily located in the nucleus and participate in regulating parental gene transcription by binding to RNA polymerase II (Pol II) complexes [21]. The circulated RNA of the sub-source is mainly located in the cytoplasm and binds to miRNAs by a competing endogenous RNAs mechanism to block the inhibition of the target gene expression [22–24]. Additionally, some endogenous circRNAs can be translated into polypeptides and proteins [25–27].

To better study the role of circRNAs in NSCLC development, we used high-throughput RNA-Sequencing (RNA-Seq) to detect differences in the expression of circRNAs in tissues of NSCLC patients, and combined bioinformatics and metabolomics analyses to explore the biological pathways in which differentially-expressed circRNAs may be involved. The data showed that circ-SEC31A was upregulated in NSCLC and that high circ-SEC31A expression predicted a poor prognosis. Mechanistic *in vitro* and *in vivo* experiments showed that silencing circ-SEC31A in NSCLC decreased tumor cell proliferation, migration, invasion, and malate-aspartate metabolism by regulating the miR-520a-5p/GOT-2 axis.

## RESULTS

### High circ-SEC31A expression predicted an unfavorable prognosis in NSCLC patients

**Figure 1:**
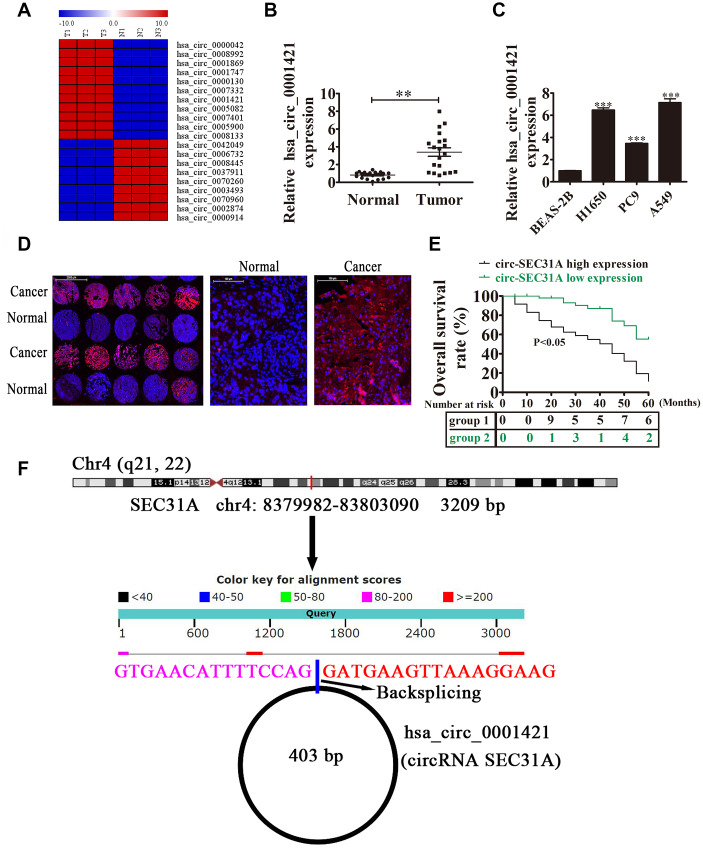
**The expression of circ-SEC31A predicted an unfavorable prognosis in non-small cell lung cancer (NSCLC) patients.** (**A**) Heat map of all differentially-expressed circRNAs between normal and tumor tissues. (**B**) RT-qPCR showing the relative expression of the circ-SEC31A from 20 NSCLC tumor tissues and adjacent non-tumor tissues. Data are presented as mean ± SD; **p<0.01. (**C**) RT-qPCR detection showing the relative expression of circ-SEC31A in A549, PC9, H1650, and normal lung epithelial cells (BEAS-2B). Data are presented as mean ± SD; ^***^P<0.001 vs. the normal group. (**D**) The expression of circ-SEC31A in NSCLC was analyzed by *in situ* hybridization on a NSCLC tissue chip (90 cases). (**E**) Prognostic significance of circ-SEC31A expression for NSCLC patients was performed with FISH values by using the median value as the cut-off; the observation time was 60 months. (**F**) The genomic loci of the *SEC31A* gene and circ-SEC31A. N, non-tumor tissues; T, tumor tissues.

To uncover the role of circRNAs in NSCLC development, the circRNA expression signatures of NSCLC tissues were explored by RNA-Seq. The data showed that circ-SEC31A was significantly upregulated in NSCLC tissues (Figure 1A). Circ-SEC31A is derived from exon 2 of the *SEC31A* gene, whose mature spliced sequence length is 403 bp. This gene is located on chromosome 4: 83799882-83803090 (Figure 1F). We also detected circ-SEC31A level by RT-qPCR in 20 paired NSCLC cancer and adjacent noncancerous lung tissues. Compared with normal tissues, circ-SEC31A was highly expressed in NSCLC tissue (Figure 1B). Furthermore, associations between circ-SEC31A expression and clinicopathological factors and the prognosis of NSCLC patients were explored (Table 1). The samples were divided into relatively high (above the adjacent normal tissues; n=50) and relatively low (below the adjacent normal tissues; n=42) levels of circ-SEC31A expression. We found no relationship between circ-SEC31A expression and clinical factors, including sex (males and females) or patient age (≤60 years and >60 years). However, there were significant differences in the circ-SEC31A expression groups regarding lymph node metastasis (negative and positive), TNM stage (I/II and III/IV), and tumor size (≤3 cm and >3 cm). Thus, high circ-SEC31A expression was associated with increased lymph node metastasis, higher TNM stage, and larger tumor size compared with the low circ-SEC31A expression group. NSCLC patients with high circ-SEC31A levels had shorter overall survival times than those with low circ-SEC31A levels according to Kaplan–Meier survival curve analysis (*p*<0.05) (Figure 1E). FISH analysis revealed that circ-SEC31A copy number was significantly increased in NSCLC tissues compared with normal tissues (Figure 1D). The level of circ-SEC31A was increased in three NSCLC cell lines (A549, PC9 and H1650), compared with normal lung epithelial cells BEAS-2B, with the highest expression levels observed in A549 and H1650 cells (Figure 1C). Consequently, A549 and H1650 were selected to evaluate the role of circ-SEC31A in further experiments.

**Table 1:** The clinic-pathological factors of 92 NSCLC patients.

**Characteristics**	**Numbers**	**hsa_circ_0001421**		***P* value**
		**Low (N = 42)**	**High (N = 50)**	
Sex				0.823
male	50	22	28	
female	42	20	22	
Age				0.923
≤50	45	21	24	
>50	47	21	26	
TNM stage				0.035
I and II	54	31	23	
III and IV	38	11	27	
Lymph node metastasis				0.021
negative	41	27	14	
positive	51	15	36	
Tumor size				0.018
≤ 3 cm	50	28	22	
> 3 cm	42	14	28	

### Downregulation of circ-SEC31A suppressed NSCLC proliferation, invasion, and migration both *in vitro* and *in vivo*

**Figure 2:**
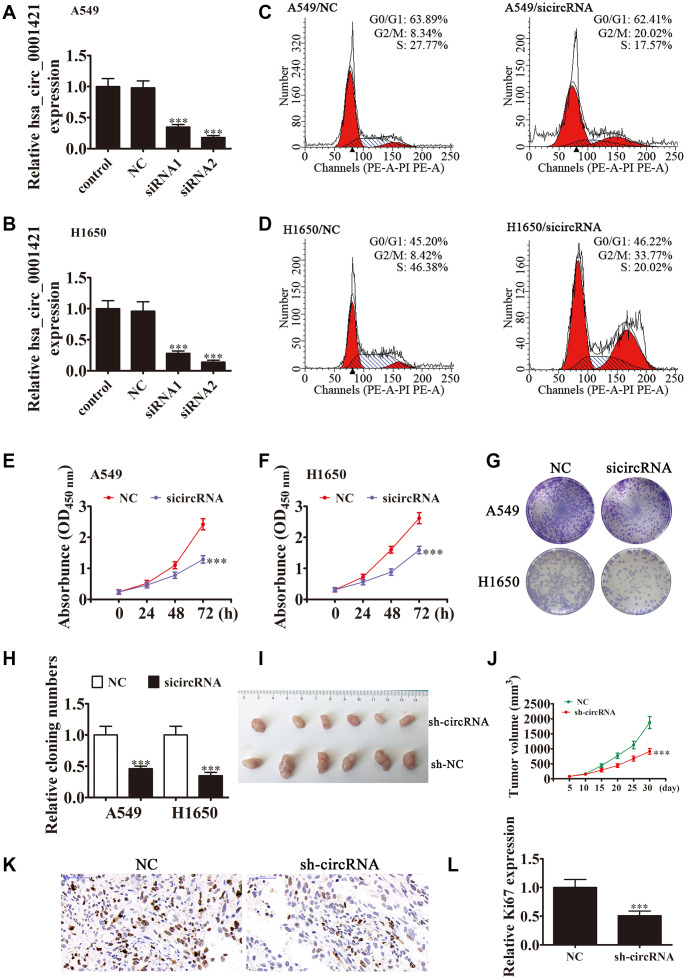
**Downregulation of circ-SEC31A suppressed NSCLC proliferation both *in vitro* and *in vivo*.** (**A** and **B**) RT-qPCR detection showing the expression of circ-SEC31A in both A649 (**A**) and H1650 (**B**) cells after transfection with siRNA against circ-SEC31A (si-circRNA) or negative control (NC). Data are presented as mean ± SD; ^***^P<0.001 vs. the normal group. (**C** and **D**) Flow cytometry detection showing the percentages of cells in G1, S, or G2 phase in both A549 (**C**) and H1650 (**D**) cells. (**E** and **F**) CCK8 assays were used to evaluate cell proliferation. Data are presented as mean ± SD; ^***^P<0.001 vs. NC. (**G** and **H**) Colony formation assay showing proliferation in both A549 and H1650 cells. Data are presented as mean ± SD; ^***^P<0.001 vs. NC. (**I** and **J**) Xenograft tumor studies. A549 cells transfected with NC or sh-circRNA were subcutaneously injected into nude mice, and tumor growth curves were plotted. Data are presented as mean ± SD; **P<0.01, ***P<0.001 vs. NC. (**K**) Immunohistochemistry showing the percentage of Ki-67-positive cells. (**L**) The relative levels Ki-67-positive cells were calculated. Data are presented as mean ± SD; ^***^P<0.001 vs. sh-NC.

**Figure 3:**
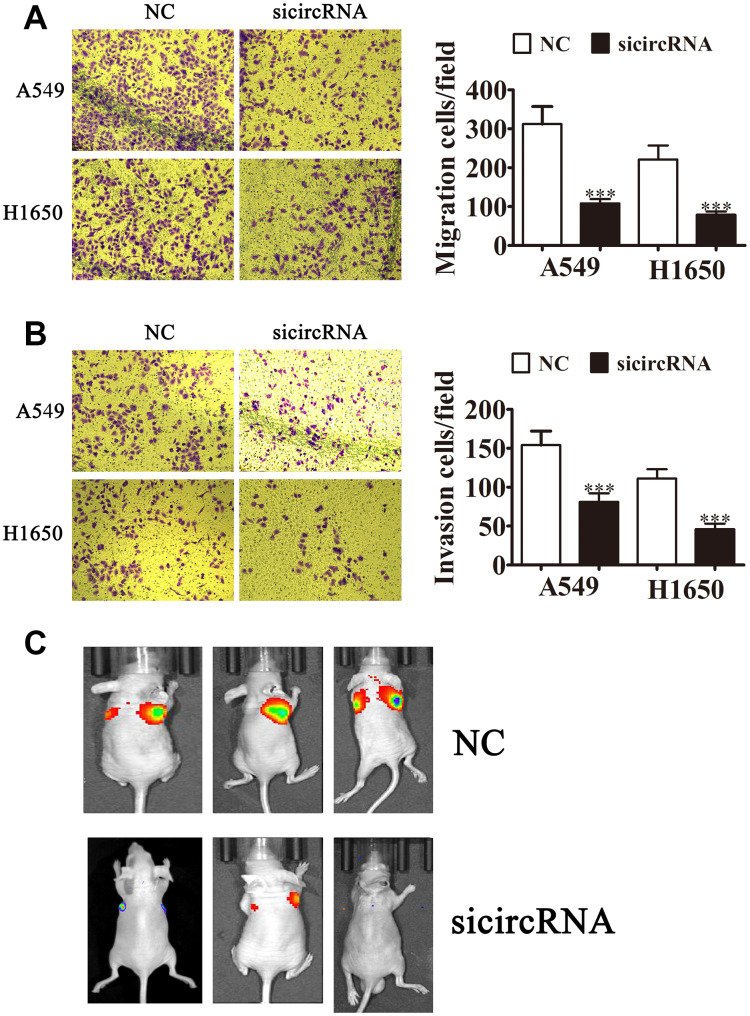
**Downregulating circ-SEC31A suppressed NSCLC invasion and migration both *in vitro* and *in vivo*.** (**A**, **B**) Cell migration (**A**) and invasion (**B**) were assessed in both A549 and H1650 cells using Transwell assays. Data are presented as mean ± SD; ^***^P<0.001 vs. NC. (**C**) Live imaging showing the effects of hsa_circ_000142 on the metastasis of A549 cells 30 d after intravenous tail injection.

To clarify the role of circ-SEC31A in NSCLC, we constructed siRNA vectors against circ-SEC31A (sicircRNA1 and sicircRNA2) to inhibit the level of circ-SEC31A; we used sicircRNA2 for subsequent studies. As expected, 48 h following transfection, RT-PCR data from both A549 and H1650 cells showed that the si-circRNA vector significantly downregulated circ-SEC31A expression (Figure 2A and 2B). The fraction of cells in G0/G1, S, and G2/M phases were calculated in Figure 2C and 2D. Si-circRNA treatment caused a significantly higher fraction of NSCLC cells in the G2/M phase (20.02% in A549 and 33.77% in H1650) than in the NC cells (8.34% in A549 and 8.42% in H1650), whereas the fraction of cells in S phase was reduced (17.57% in A549 and 20.02% in H1650) compared with NC cells (27.77% in A549 and 46.38% in H1650), suggesting that downregulating circ-SEC31A might induce a G2/M arrest in NSCLC cells. CCK8 assays showed that downregulation of circ-SEC31A inhibited the growth of A549 and H1650 cells (Figure 2E and 2F). Furthermore, colony formation assays indicated that si-circRNA resulted in a significant reduction in colony numbers compared with the NC groups in A549 and H1650 cells (Figure 2G and 2H). To determine if silencing circ-SEC31A suppressed tumor growth in nude mice, we constructed stably-transfected A549 cells with circ-SEC31A knockdown and established a xenograft mouse model in which an equal number of A549 cells were injected into the mice (n = 6). The results showed that silencing circ-SEC31A significantly reduced tumor volume compared with the NC group (Figure 2I and 2J). Ki67 is a marker of cell proliferation, and the number of proliferating cells evaluated by Ki67 immunohistochemistry was lower in the sh-circRNA group (Figure 2K and 2L). Transwell results also showed that silencing circ-SEC31A inhibited the migration and invasion abilities of A549 and H1650 cells (Figure 3A and 2B). Furthermore, we used a small animal live imaging system to obtain fluorescence images of nude mice 30 d after tail-vein inoculation (Figure 3C). The fluorescence intensity in the si-circRNA group was consistently weaker than the NC group.

### Metabolic analysis showed the effect of circ-SEC31A on NSCLC cell metabolism

**Figure 4:**
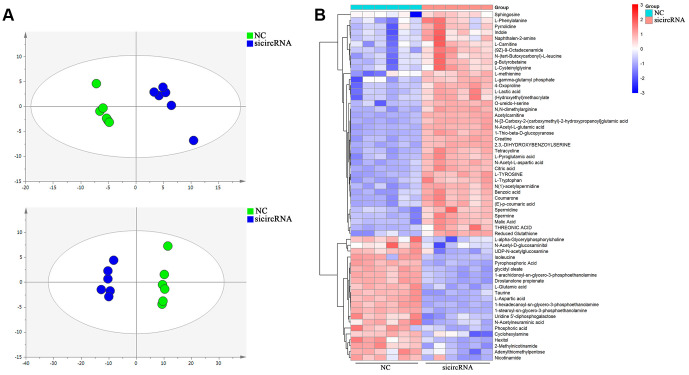
**Cell metabolites analysis showing the effect of circ-SEC31A on NSCLC cell metabolites.** (**A**) OPLS-DA score plot showing the difference between the si-circRNA and NC groups of A549 cells. (**B**) Heatmap identifying metabolites in A549 cells. Colors in the heatmap change with the metabolite contents, red indicates high content, while blue indicates low content.

**Figure 5:**
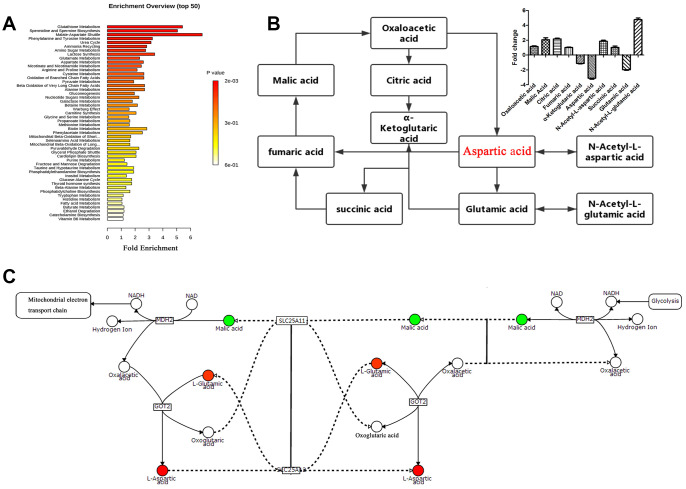
**Pathway analysis of the differentially-expressed proteins.** (**A**) Pathway analysis of significantly altered metabolites in A549 cells compared with baseline. (**B** and **C**) Compound reaction networks of the metabolites and genes were visualized using Metscape: genes (circles), metabolic enzymes (squares) are presented as nodes, and relationships are presented as edges. Input genes are shown in blue, input metabolites are shown in red. The metabolite-gene association network was primarily related to malate-aspartate metabolism.

Score plots of OPLS-DA in A549 cells (Figure 4A) revealed a strong separation of the si-circRNA and NC groups in both positive and negative mode. Different metabolites were examined by OPLS-DA and variable importance in projection (VIP>1) and t-test (*p*<0.05) were used for screening. Finally, 138 metabolites in A549 cells were found, 26 of which were identified by searching the library (Figure 4B). We discovered many markedly changed pathways in the solasodine groups at the metabolomic level, including Glutathione Metabolism, Spermidine and Spermine Biosynthesis, the Malate-Aspartate Shuttle, Urea Cycle, Phenylalanine and Tyrosine Metabolism (Figure 5A). Compound reaction networks of the metabolites and genes were visualized using Metscape Analysis Software and the KEGG database. The results showed that Aspartic acid and GOT2 were significantly downregulated after silencing circ-SEC31A (Figure 5B and 5C).

### The relationships among miR-520a-5p, circ-SEC31A, and GOT2

**Figure 6:**
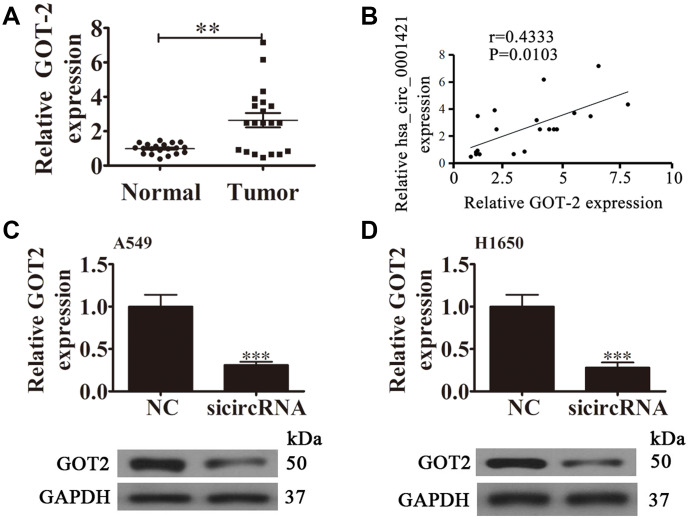
**The relationship between circ-SEC31A and GOT2.** (**A**) RT-qPCR showing the relative GOT2 expression from 20 NSCLC tumor tissues and adjacent non-tumor tissues. Data are presented as mean ± SD; **p<0.01. (**B**) A significant positive correlation between circ-SEC31A and GOT2 was detected in NSCLCs tissues; n=20, P=0.0103. (**C** and **D**) RT-qPCR and western blot detection showing the expression of GOT2 in both A549 (C) and H1650 (D) cells. Data are presented as mean ± SD; ^***^P<0.001 vs. NC.

**Figure 7:**
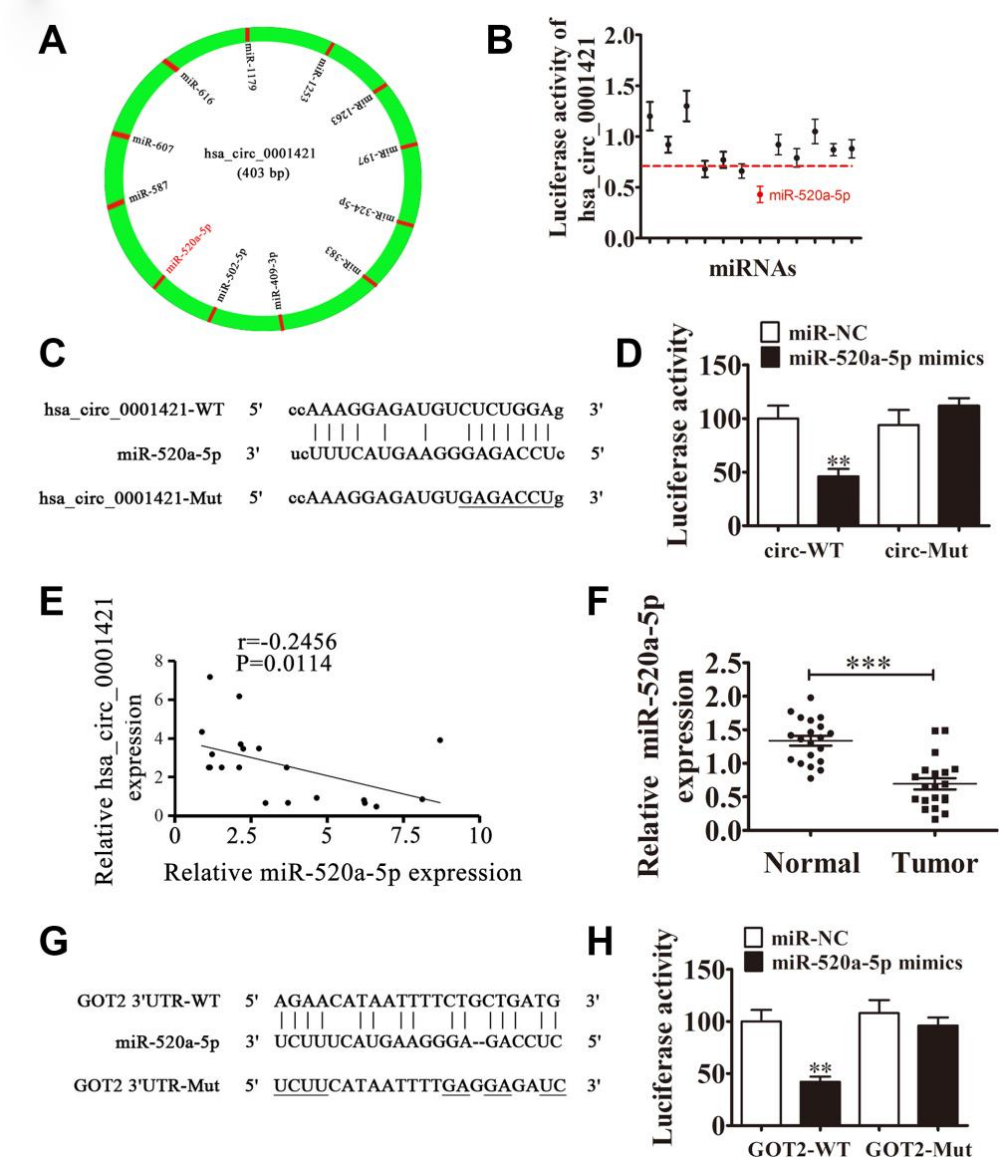
**The relationships among miR-520a-5p, circ-SEC31A, and GOT2.** (**A**) A schematic model showing the putative binding sites of 12 predicted miRNAs on circ-SEC31A. (**B**) Luciferase activity of circ-SEC31A in HEK293T cells transfected with miRNA mimics, which putatively bind to the circ-SEC31A sequence. Luciferase activity was normalized to Renilla luciferase activity. (**C**) The predicted binding sites of miR-520a-5p in circ-SEC31A. The mutated (Mut) version of circ-SEC31A is also shown. (**D**) Relative luciferase activity was determined 48 h after transfection with miR-520a-5p mimic/normal control (NC) or with the circ-SEC31A wild-type/Mut in HEK293T cells. Data are presented as mean ± SD; ^***^P<0.001. (**E**) A significant negative correlation between circ-SEC31A and miR-520a-5p was detected in NSCLCs tissues; n=20, P=0.0114. (**F**) RT-qPCR showing the relative expression of miR-520a-5p from 20 NSCLC tumor tissues and adjacent non-tumor tissues. Data are presented as mean ± SD; ^***^P<0.001. (**G**) The predicted binding sites of miR-520a-5p within the 3′-UTR of GOT2. The mutated version of the 3′-UTR of GOT2 is also shown. (**H**) Relative luciferase activity was determined 48 h after transfection with miR-520a-5p mimic/normal control or with the 3′-UTR-GOT2 wild-type/Mut in HEK293T cells. Data are presented as mean ± SD; ^***^P<0.001.

**Figure 8:**
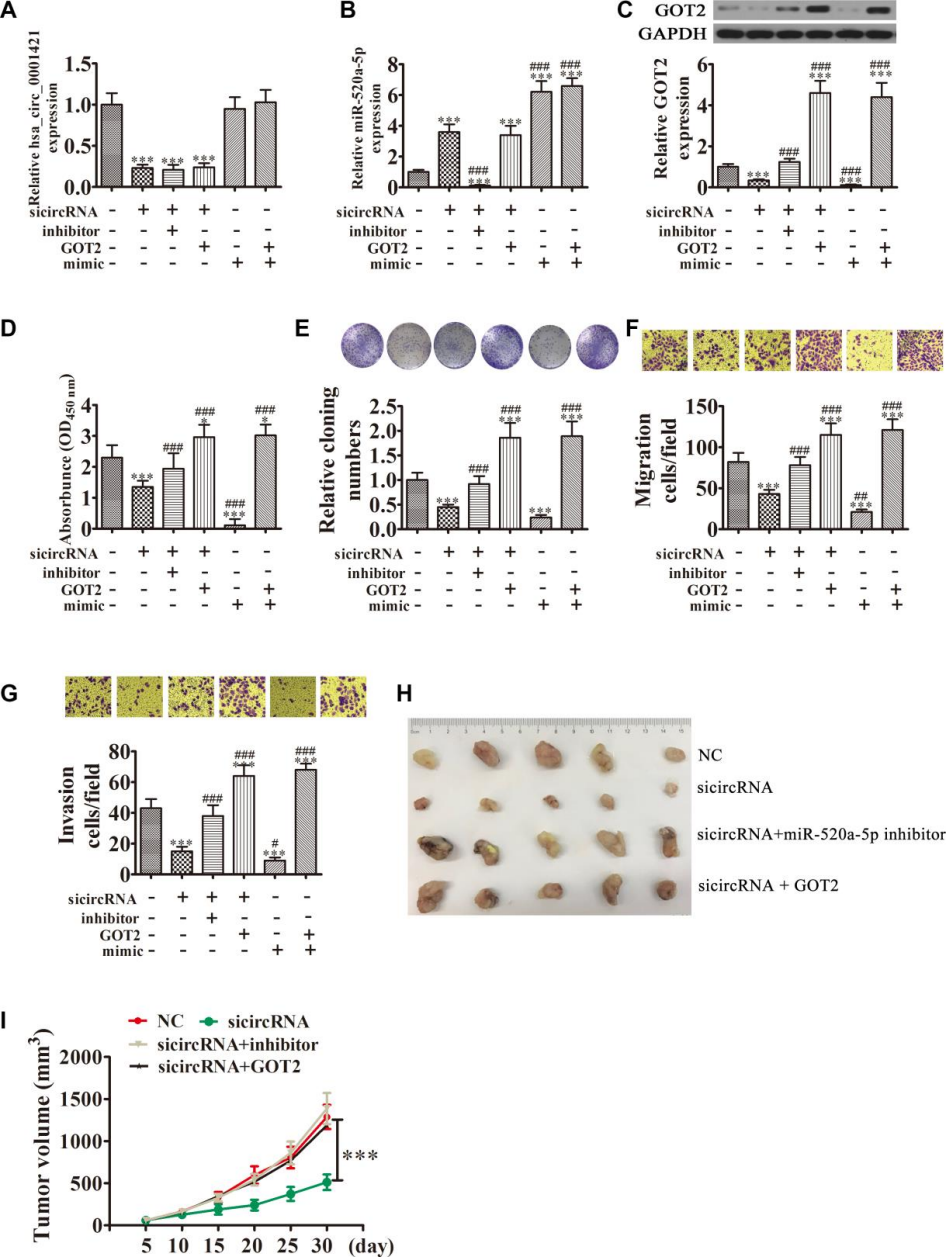
**Overexpression of miR-520a-5p or downregulation of GOT2 reversed the suppressive effect of circ-SEC31A silencing on NSCLC cell proliferation, invasion, and migration.** (**A** and **B**) RT-qPCR detection showing the expression of circ-SEC31A and miR-520a-5p in A649 cells. Data are presented as mean ± SD; ^***^P<0.001 vs. NC; ^###^P<0.001 vs. si-circRNA. (**C**) Western blot analysis showing the expression of GOT2 in A649 cells. Data are presented as mean ± SD; ^***^P<0.001 vs. NC; ^###^P<0.001 vs. si-circRNA. (**D**) CCK8 assays were used to evaluate cell proliferation after 72 h culture. Data are presented as mean ± SD; ^*^P<0.05, ^***^P<0.001 vs. NC; ^###^P<0.001 vs. si-circRNA. (**E**) Colony formation assays showing the proliferation of A549 cells. Data are presented as mean ± SD; ^***^P<0.001 vs. NC; ^###^P<0.001 vs. si-circRNA. (**F** and **G**) Cell migration (**E**) and invasion (**F**) were assessed in A549 cells using Transwell assays. Data are presented as mean ± SD; ^***^P<0.001 vs. NC; ^#^P<0.05, ^##^P<0.01, ^###^P<0.001 vs. si-circRNA. (**G**) Xenograft tumors in nude mice from the four treatment groups (NC, si-circRNA, si-circRNA + miR-520a-5p inhibitor, si-circRNA + GOT2) after subcutaneous injection of A549 cells. (**H, I**) Xenograft tumor volumes from the four treatment groups were measured at the indicated time points. Data are presented as mean ± SD; ***P<0.001.

RT-qPCR analysis also found that GOT2 expression was increased in NSCLC tissues compared with adjacent normal tissues (Figure 6A). A positive correlation between circ-SEC31A expression and GOT2 levels was observed in NSCLC tissues (Figure 6B). RT-qPCR and western blot analyses showed that downregulating circ-SEC31A decreased the protein and mRNA levels of GOT2 in A549 and H1650 cells (Figure 6C and 6D). To further study the mechanism of circ-SEC31A, bioinformatics analysis (https://circinteractome.nia.nih.gov/) was used to select potential miRNA targets with shared common binding sites for circ-SEC31A (Figure 7A). We designed a circ-SEC31A luciferase reporter screen for these miRNAs. We found that miR-520a-5p reduced the luciferase activity from the circ-SEC31A luciferase reporter by at least 80% (Figure 7B). These results revealed that miR-520a-5p had a conserved binding site for circ-SEC31A (Figure 7C). A dual-luciferase reporter assay was consequently performed in HEK293T cells. Wild-type and mutant circ-SEC31A sequences were cloned to construct the reporter plasmids and mutant vectors, respectively. While it was found that co-transfection of the miR-520a-5p mimics with the reporter plasmids decreased luciferase activity, conversely, co-transfection of miR-520a-5p mimics and mutated vectors showed no significant changes in luciferase activity. Hence, these data proved that miR-520a-5p is a direct target of circ-SEC31A (Figure 7D). A negative correlation between circ-SEC31A expression and miR-520a-5p levels was also observed in NSCLC tissues (Figure 7E). RT-qPCR detection also found that miR-520a-5p expression was decreased in NSCLC tissues compared with adjacent normal tissues (Figure 7F). Next, bioinformatics analysis (http://www.targetscan.org/) showed that GOT2 was a potential target of miR-520a-5p. To confirm that GOT2 is a target of miR-520a-5p, the wild-type and mutant GOT2 sequences were cloned to construct reporter plasmids and mutant vectors, respectively (Figure 7G). These data revealed that, while co-transfection of miR-520a-5p mimics and reporter plasmids visibly suppressed luciferase activity, co-transfection of miR-520a-5p mimics and mutated GOT2 vectors had no significant effect on luciferase activity. Hence, these results prove that miR-520a-5p directly targets GOT2 (Figure 7H). Next, RT-qPCR results showed that circ-SEC31A levels were unchanged after transfecting the miR-520a-5p inhibitor (inhibitor), miR-520a-5p mimic, or overexpressing GOT2 (GOT2), compared with the control groups in A549 cells (Figure 8A). Levels of miR-520a-5p were significantly increased after transfecting si-circRNA or miR-520a-5p mimic, while they were decreased after transfecting the miR-520a-5p inhibitor in A549 cells (Figure 8B). RT-qPCR and western blot showed that si-circRNA and miR-520a-5p mimic decreased the protein and mRNA levels of GOT2, while the miR-520a-5p inhibitor or GOT2 overexpression increased GOT2 in A549 cells (Figure 8C). CCK8 assays showed that si-circRNA and miR-520a-5p mimic suppressed the growth of A549 cells, while the miR-520a-5p inhibitor or GOT2 overexpression promoted growth (Figure 8D). Colony formation assays using A549 cells confirmed these results (Figure 8E). Furthermore, Transwell results showed that si-circRNA and miR-520a-5p mimic inhibited A549 cell migration and invasion, while miR-520a-5p inhibitor or GOT2 overexpression promoted migration and invasion (Figure 8F and 8G). The *in vivo* xenograft mouse models using A549 cells also showed that downregulating miR-520a-5p or upregulating GOT2 restored the *in vivo* tumor growth ability of A549 cells after circ-SEC31A knockdown (Figure 8H and 8I). Together, these findings suggest that miR-638/GOT2 is a downstream target of circ-SEC31A, and that circ-SEC31A promotes NSCLC progression by sponging miR-520a-5p, which increases GOT2 expression.

## DISCUSSION

Recently, circRNAs have been found to be involved in the development of various diseases, including arteriosclerosis [28], nervous system disorders [29, 30], aging [31, 32], heart failure [33, 34], and malignant tumors [35, 36]. In NSCLC, circRNAs have also been shown to play important roles in tumor growth, apoptosis, and metastasis [37–39]. In this study, we found a novel circRNA (hsa_circ_0001421, circ-SEC31A) that was increased in human NSCLC tissues by high-throughput RNA-Seq. We also found 20 patients in which we confirmed the association between circ-SEC31A and NSCLC by RT-qPCR. Moreover, our study revealed that decreasing circ-SEC31A could limit cell proliferation and stimulate apoptosis, as well as suppress NSCLC growth *in vitro* and *in vivo*. Nevertheless, various studies have reported that NSCLC patients bearing *KRAS* mutations not only have a significantly better prognosis than their *KRAS*-wild type counterparts, but typically present with low survival rates [40, 41]. Hence, we will confirm associations between *KRAS* mutational status and circ-SEC31A levels in NSCLC patients in future studies.

MiRNAs are a class of small non-coding RNAs that are associated with the inhibition or degradation of mRNAs by binding to complementary sequences in target messages. MiRNAs play a central role in various biological processes, including cell differentiation, colonization, and apoptosis [42]. With the gradual development of research in this field, miRNAs are more frequently being found to play key roles in NSCLC development either through the direct regulation of various NSCLC signaling pathways or of the microenvironment, which regulates tumor growth [43–45]. As the nucleic acid sequences of the miRNAs and target regions do not have to be perfectly complementary, miRNAs can simultaneously regulate the expression of hundreds of genes [46]. Aberrant miR-520a-5p levels have been found in many diseases, including psoriasis [47] and cardiomyocyte injury [48], but miR-520a-5p has controversial roles in the development of various human cancers. Previous studies of colorectal cancer [49], hepatocellular carcinoma [50, 51], and chronic myelogenous leukemia [52] have found that miR-520a-5p inhibits tumor development. Many studies have shown that circRNAs could play a role as sponges that absorb miRNAs [11]. Our study analyzed the regulatory mechanism of circ-SEC31A at the post-transcriptional level. Using bioinformatics, we found that the circ-SEC31A 3′-UTR shares identical miR-520a-5p response elements and could competitively bind to miR-520a-5p. These results indicated that circ-SEC31A could act as competitive endogenous RNAs to sponge miR-520a-5p during NSCLC progression.

Aspartate is essential for cell cycle progression, and it is the most essential intermediary for amino acid, purine nucleotide, and pyrimidine nucleotide synthesis [53]. Glutamate Oxaloacetate Transaminase (GOT) is also known as aspartate aminotransferase. There are two kinds of GOT in eukaryotes, GOT1 and GOT2 [54]. The involvement of GOT2 in cancer cell metabolism is mainly reflected in the following three aspects: (1) GOT2 catalyzes the formation of glutamic acid and oxaloacetate from aspartic acid and α-glutaric acid, which participate in the Krebs cycle and may provide energy for tumor cells; (2) GOT2 is a key enzyme in the transfer of malate-aspartate in glycolysis and participates in the amino acid metabolism of tumor cells [55, 56]. Finally, (3) GOT2-derived glutamate that is produced from aspartic acid can be converted into glutamine. According to previous studies, glutamine is a transitional demand for growth of tumor cells, which can have the characteristic of glutamine addiction [57]. Son et al. found that after reducing GOT2 activity and cutting off the reaction pathway of intracellular glycolysis and TCA, pancreatic cancer and breast cancer cells will undergo dystrophic metabolism and acquire glutamine restriction, thereby inhibiting proliferation [58]. Metabolomics analysis of NSCLC cells were performed to verify the mechanism of circ-SEC31A. OPLS-DA and Metscape were applied to assess the metabolomics data. Our results showed that GOT2 had abnormal expression in the aspartic acid pathway. Dual-luciferase reporter assays confirmed that miR-520a-5p interacted with the 3′-UTR of GOT2, inhibiting GOT2 at the post-transcriptional level. Furthermore, we found that GOT2 overexpression promoted A549 cell proliferation, migration, and invasion.

## CONCLUSIONS

This study indicated that circ-SEC31A plays an important role in regulating NSCLC cell proliferation, migration, invasion, and malate-aspartate metabolism by sponging miR-520a-5p, verifying it as a promising prognostic biomarker in NSCLC. Finally, we discovered that the circ-SEC31A/miR-520a-5p/GOT2 axis is a potential therapeutic target for NSCLC treatment.

## MATERIALS AND METHODS

### Tissue samples

In total, 92 fresh NSCLC tissues and paired adjacent noncancerous lung tissues were collected after obtaining informed consent from patients at Renji Hospital of Shanghai Jiaotong University, China. The data were censored at the last follow-up visit or at the time of the patient’s death without relapse. Histological and pathological diagnostics for NSCLC were evaluated based on the Revised International System for Staging Lung Cancer (this revised system has been used since 1986 and includes modifications to the rules for staging the tumor, node, metastasis (TNM) anatomic subsets [45], more specific staging categories and consistency for reporting the end results for Stage I, Stage II, and Stage IIIA disease are also provided). Patients received neither chemotherapy nor radiotherapy before tissue sampling (Table 1). The samples were snap-frozen in liquid nitrogen and stored at −80 °C prior to RNA extraction. This study was approved by the Ethics Committee of Renji Hospital at Shanghai Jiaotong University.

### Strand-specific RNA-Seq library construction and high-throughput RNA-Seq

Total RNA was extracted from three paired NSCLC tissues and adjacent noncancerous lung tissues using TRIzol Reagent (Invitrogen, Carlsbad, CA, USA). Approximately 3 μg of total RNA from each sample was subjected to the VAHTS Total RNA-seq (H/M/R) Library Prep Kit from Illumina (Vazyme Biotech Co., Ltd, Nanjing, China) to remove ribosomal RNA while retaining other types of RNA, including mRNA and ncRNA. Purified RNA was treated with RNase R (Epicenter, 40 U, 37 °C for 3 h), followed by purification with TRIzol. RNA-seq libraries were prepared using the KAPA Stranded RNA-Seq Library Prep Kit (Roche, Basel, Switzerland) and subjected to deep sequencing with an Illumina HiSeq 4000 at Aksomics, Inc., Shanghai, China (Accession code: H1712024).

### Cell lines and culture

The normal human lung epithelial cell line, BEAS-2B, and the NSCLC cell lines, A549, PC9, and H1650 were obtained from the Cell Bank of the Chinese Academy of Sciences and cultured in Dulbecco’s Modified Eagle’s Medium (Life Technologies, Carlsbad, CA, USA) supplemented with 100 IU/mL penicillin, 100 μg/mL streptomycin, and 10% fetal bovine serum (FBS; Invitrogen) at 37 °C in a humidified atmosphere with 5% CO_2_.

### Fluorescence *in situ* hybridization (FISH)

Specific probes for circ-SEC31A (Dig-5′-CCTTTAACTTCATCCTGGAAAATGTTCACA-3′-Dig) were prepared by Geneseed Biotech (Guangzhou, China). Signals were detected by Cy3-conjugated anti-digoxin and FITC-conjugated anti-biotin antibodies (Jackson ImmunoResearch Inc., West Grove, PA, USA). Nuclei were counterstained with 4,6-diamidino-2-phenylindole (DAPI). Finally, images were obtained on a Zeiss LSM 700 confocal microscope (Carl Zeiss, Oberkochen, Germany).

### Bioinformatics analysis

The circRNA/miRNA target genes were predicted using the website, https://circinteractome.nia.nih.gov/. The interactive relationship between miR-520a-5p and GOT-2 was predicted using the website, http://www.targetscan.org/.

### Total RNA isolation and quantitative reverse transcription polymerase chain reaction (RT-qPCR)

Total RNA was isolated from tumor tissues or cells using TRIzol reagent (Invitrogen), following the manufacturer’s protocol. The purity and concentration of RNA samples were examined spectrophotometrically by measuring absorbance at 260 nm, 280 nm, and 230 nm with a NanoDrop ND-1000 (Thermo Fisher Scientific, Wilmington, DE, USA). Specifically, OD260/OD280 ratios between 1.8 and 2.1 were deemed acceptable, and OD260/OD230 ratios >1.8 were also deemed acceptable.

Total RNA was reverse transcribed before RT-qPCR detection. Primers specific for circ-SEC31A, miR-520a-5p, and GOT-2 were obtained from GenePharma (Shanghai, China). RT-qPCR was performed using an AB7300 thermo-recycler (Applied Biosystems, Carlsbad, USA) with primers and TaqMan Universal PCR Master Mix. *GAPDH* was used as the reference gene for circRNAs and mRNAs. U6 was used as an internal control for the level of miRNA expression. Gene expression was quantified using the 2^−ΔΔCt^ method. The primers used to assay *circ-SEC31A* expression included forward, 5′-TCTCTGGAGTTCTGATTGCAGGTGG-3′ and reverse, 5′-TGCTAGGTAAATGGGGTGATTCTGG-3′. The *miR-520a-5p* primers were forward, 5′-ACACTCCAGCTGGGCTCCAGAGGG-3′ and reverse, 5′-CTCAACTGGTGTCGTGGAGTCGGCAATTCAGTTGAGAGTTTGTAC-3′. The *GOT-2* primers were forward, 5′-GAGCAGGGCATCAATGTCTG-3′ and reverse, 5′-GTTGGAATACAGGGGACGGA-3′. The *U6* primers were forward, 5′-CTCGCTTCGGCAGCACA-3′ and reverse, 5′-AACGCTTCACGAATTTGCGT-3′. The *GAPDH* primers were forward, 5′-GCACCGTCAAGGCTGAGAAC-3′ and reverse, 5′-GGATCTCGCTCCTGGAAGATG-3′.

### RNA interference and overexpression

The miR-520a-5p inhibitors (5′-CUCCAGAGGGAAGUACUUUCU-3′), miR-520a-5p mimics (5′-CUCCAGAGGGAAGUACUUUCU-3′), and siRNA against circ-SEC31A (5′-CCGGCTCTGGAGTTCTGATTGCATTCTCGAGTGCAATCAGAACTCCAGAGTTTTTTTG-3′) were purchased from GenePharma. Transfections were performed in accordance with the supplier's protocol. Briefly, cells were transferred to 6-well culture plates and transfected using Lipofectamine 2000 (Invitrogen). To induce GOT-2 overexpression, a pCDNA3.0 vector was transfected, as described above. For xenograft experiments, lentiviral-mediated circ-SEC31A-silencing (si-circRNA) A649 were constructed.

### Dual-luciferase reporter assay

The binding site of circ-SEC31A and the 3'-UTR of GOT-2, termed circ-SEC31A-WT, circ-SEC31A-Mut, GOT-2-3'UTR WT, and GOT-2-3'UTR-Mut were inserted into the KpnI and HindIII sites of the pGL3 promoter vector (Realgene, Nanjing, China) in a dual-luciferase reporter assay. First, cells were plated into 24-well plates. Then, 80 ng plasmid, 5 ng Renilla luciferase vector pRL-SV40, 50 nM miR-520a-5p mimics, and negative control were transfected into cells with lipofectamine 2000 (Invitrogen). The cells were then collected and measured 48-h after transfection using a Dual-Luciferase Assay (Promega, Madison, WI, USA), following the manufacturer’s instructions. All experiments were independently repeated three times.

### Cell proliferation assay

The Cell Counting Kit-8 (CCK-8) assay was used to detect cellular proliferation. Transfected cells were seeded into 96-well plates at a density of 5,000 cells/well in triplicate. Cell viability was measured using the CCK-8 system (Gibco) at 0, 24, 48, 72, and 96 h after seeding, according to the manufacturer’s instructions.

For colony formation assays, transfected cells were seeded into six-well plates at a density of 2,000 cells/well and maintained in DMEM containing 10% FBS for 10 d. Colonies were imaged and counted after they were fixed and stained.

### Transwell migration assay

Cell migration was analyzed using Transwell chambers (Corning Inc., Corning, NY, USA) in accordance with the manufacturer’s protocol. After incubation for 24 h, cells on the upper surfaces of Transwell chambers were removed using cotton swabs, and cells located on lower surfaces were fixed with methanol for 10 min, followed by crystal violet staining. Stained cells were imaged and counted in five randomly selected fields. In invasion experiments, chamber inserts were coated with 200 mg/mL Matrigel and dried overnight under sterile conditions.

### Flow cytometry analysis of cell cycle progression

Cells were fixed in 70% ethanol overnight at 4°C. Then, fixed cells were resuspended in staining solution (Beyotime, Shanghai, China) and incubated for 30 min at 4 °C. Finally, stained cells were measured by flow cytometry (Beckman Coulter, Franklin Lakes, NJ, USA).

### Animal studies

To examine the role of circ-SEC31A in a lung cancer metastasis model, 1×10^6^ stable lentiviral-mediated circ-SEC31A-silenced (si-circRNA) or negative control (si-NC) A549 cells were intravenously injected into male nude mice through the tail vein (Chinese Science Academy, Shanghai, China). After a month, lung metastases were measured and quantified using an *in vivo* bioluminescent imaging with an IVIS Lumina series III *in vivo* Imaging System (PerkinElmer, New York, NY, USA).

For xenograft assays, 1×10^6^ modified (circ-SEC31A downregulation, circ-SEC31A downregulation+miR-520a-5p inhibitor, and circ-SEC31A downregulation+GOT2 overexpression) or control (wild-type) A549 cells were injected subcutaneously into the right side of each male nude mouse (Chinese Science Academy). Tumor volumes (length × width^2^ × 0.5) were measured at the indicated time points, and tumors were excised 4 weeks after injection.

For metastasis analysis, wild-type or circ-SEC31A downregulation A549 cells (2×10^5^) were transfected with luciferase expression vectors, and the cells were injected intravenously into the tails of mice. After 30 d, A549 cell metastasis was analyzed by bioluminescence imaging following an intravenous injection of luciferin (150 mg luciferin/kg body weight) into the tails.

All mice were maintained and handled according to protocols approved by the Animal Care Committee of Renji Hospital of Shanghai Jiaotong University.

### Immunohistochemistry

Tumor tissue samples were fixed in a 10% formalin and embedded in paraffin. Sections (5 μm-thick) were stained with Ki67 to evaluate proliferation. Sections were examined using an Axiophot light microscope (Zeiss) and imaged with a digital camera.

### Metabolomics data collection and analysis

A549 cells with or without silencing circ-SEC31A were used for metabolomics analyses. Briefly, cells were plated into 6-well plates at an initial density of 1×10^6^ per well. After culture, the supernatant was discarded, and each well was washed with PBS three times. Then 2 mL of 4 °C methanol was added, and cells were scraped. Cells were lysed with a cell pulverizer to fully extract metabolites. Finally, the supernatant was centrifuged at 14000 ×g for 10 min before analysis. LC/MS data were processed by Compound Discoverer 2.1 software (Thermo Company). Principal component analysis (PCA) and orthogonal partial least squares discriminant analysis (OPLS-DA) were performed using SMICA-P 14.0 software. Metabolites were identified according to accurate masses and product ion spectra, and pathway analysis was performed using MetaboAnalyst 4.0.

### Statistical analysis

Statistical significance was evaluated by one-way analysis of variance followed by the Tukey–Compare all pairs of columns. A Pearson’s correlation test was used to determine the association between two groups using GraphPad Prism 5.02 software (GraphPad Inc., San Diego, CA, USA). The cumulative recurrence and survival rates were analyzed using Kaplan-Meier’s method. Results are presented as the mean ± SEM. P-values <0.05 were considered significant.

### Ethics approval

The study has been examined and certified by the Ethics Committee of Renji Hospital of Shanghai Jiaotong University, and informed consent was obtained from all participants included in the study, in agreement with institutional guidelines.
